# Back to the Future: Multiparent Populations Provide the Key to Unlocking the Genetic Basis of Complex Traits

**DOI:** 10.1534/genetics.117.203265

**Published:** 2017-06-06

**Authors:** Dirk-Jan de Koning, Lauren M. McIntyre

**Affiliations:** 1 Deputy Editor, G3: Genes | Genomes | Genetics Swedish University of Agricultural Sciences @DJ_de_Koning; 2 Senior Editor, GSA Journals University of Florida @LaurenMMcIntyr1

In the past decade, the ability to generate whole-genome sequences has provided geneticists with a view of the astonishing breadth of genetic variation. This, in theory, means we should be able to identify the specific differences in DNA sequence that lead to an inherited phenotype, including disease states. But this wealth of new information has revealed perhaps the most fundamental challenge for geneticists since Mendel. While we understand that phenotypes are influenced by genetic variation, we do not yet know how to interpret individual genome sequences, and, therefore, we cannot predict which genetic variants are linked to which phenotypes. Indeed, the term “missing heritability” was coined to highlight the fact that in natural populations the genes or genetic elements associated with complex traits explain only a small proportion of the phenotypic variation in these traits.

In stark contrast, controlled crosses of model organisms have generated a wealth of information about the genetic basis of phenotypes. From broad associations of genomic regions with traits, to individual polymorphisms that act by well understood mechanisms, geneticists have been remarkably successful in revealing the impact of genetic variation on phenotype. Applications as diverse as targeted drug therapy and dramatic improvements in agricultural output have been enabled by our understanding of genetics. But it remains a significant challenge to transfer this understanding to natural populations.

To bridge the gap between natural populations and experimental systems, experimental systems need to incorporate more of the complexity of natural populations. This has given rise to a burst of creativity in the design of genetic reference populations. The basic idea is simple: combine the strength of the experimental system, where the genetic composition can be replicated, with the genetic diversity of the target population. Rather than choose two inbred lines or two phenotypically divergent individuals as founders of a genetic reference panel (recombinant inbreds), choose eight, or 25. Using multiple lines as founders of a set of inbred lines whose haplotypes can be replicated has been referred to as Interconnected populations multi-parent, advanced-generation inter-cross design, Complex Cross, and multi-parental RIL. We are choosing to refer to this broad set of genetic reference panels as multi-parent populations (MPP).

Fifteen years ago, the mouse genetics community embraced the challenge of creating strains that would represent the diversity of natural variation in mice, thereby improving the utility of the organism for exploring complex human disease. Eight founder mouse strains were selected, and offspring populations with all eight haplotypes were developed in a funnel mating scheme (Figure 1, Collaborative Cross Consortium 2012). The first set of papers describing these strains was published in *GENETICS* and *G3* in 2012 (http://www.g3journal.org/content/mpp_mouse#cc). Systematic monitoring of progress with the mouse collaborative cross has provided a window into the impact of drift on the genomes (Srivastava *et al.* 2017), a startling insight into the genetic basis of male sterility (Odet *et al.* 2015; Shorter *et al.* 2017), the impact of structural variation (Morgan *et al.* 2017), and a new method for estimating haplotypes and preserving uncertainty (Oreper *et al.* 2017). The resources developed for mouse enable detection of many types of loci, from those associated with SARS (Gralinski *et al.* 2017) and West Nile (Green *et al.* 2017) virus infections to those associated with fertility (Shorter *et al.* 2017) allergens Kelada (2016). Morgan *et al.* (2016) and Dumont *et al.* (2017) also provide insights into genome structure.

This large effort in mouse is matched by ambitious projects on a plethora of organisms. MPPs have been created in plants [*Arabidopsis* (Kover *et al.* 2009), Maize (Yu *et al.* 2008), wheat (Mackay *et al.* 2014), sunflower (Bowers *et al.* 2012), and other crops (Brenton *et al.* 2016; Nice *et al.* 2016)], in animals [*Drosophila* (Mackay *et al.* 2012; King *et al.* 2012)], and in yeast (Cubillos *et al.* 2013). In 2014, we highlighted the diversity of MPPs in *GENETICS* and *G3* with articles on maize, sorghum, wheat, triticale, *Arabidopsis*, *Drosophila*, and mouse (http://www.genetics.org/content/multiparental_populations). These issues of *GENETICS* and *G3* feature MPPs of sorghum (Bouchet *et al.* 2017), strawberry (Mangandi *et al.* 2017), rice (Raghavan *et al.* 2017), oil palm (Tisné *et al.* 2017), yeast (Cubillos *et al.* 2017), *Drosophila* (King and Long 2017; Najarro *et al.* 2017; Stanley *et al.* 2017) and mouse (Gralinski *et al.* 2017; Green *et al.* 2017; Morgan *et al.* 2017; Oreper *et al.* 2017; Shorter *et al.* 2017; Srivastava *et al.* 2017; Tyler *et al.* 2017).

*GENETICS* and *G3* are committed to fostering discussion about the genetic inferences made from MPPs, as well as the best ways to analyze the data, and to extending inferences to natural populations. Projects that rely on a common set of germplasm (or set of strains) rely on data sharing. One of the benefits to working with a reference panel is the ability to leverage data collected in different ways, for different purposes. Our journals have long had policies for reagent and data sharing that reflect the values of our community, and this is evident in these articles on MPPs. Each MPP paper in these issues has the *Data availability* section that is standard for all GSA publications, as well as a one-page guide to the data that makes it easier to browse the data behind the papers.

In recognition of the ongoing importance of MPPs for understanding fundamental questions in genetics, *G3* and *GENETICS* have designed a special web resource for MPPs. Papers are organized in a special collections page, with subheaders that help navigate the growing literature. Our journals have long partnered with model organism databases FlyBase, SGD, WormBase, and others, and we now incorporate news, blogs, tips, and protocols directly on our webpage to help geneticists interested in MPPs get a handle on this topic. Tweet your insights to #MPP #GSAjournals, and use MPP as a keyword of your MPP papers to enable text search engines to collate this literature. The GSA journals are committed to creating a community platform that spans species and disciplines, yet remains focused on common research questions. We thank the authors, referees and editors for making this resource a reality!
